# Remission of type 2 diabetes: Perspectives of dietitians in Kuwait

**DOI:** 10.1371/journal.pone.0276679

**Published:** 2022-10-27

**Authors:** Dalal Alsaeed, Nicola Guess, Ebaa Al Ozairi

**Affiliations:** 1 Clinical Care Research and Trials, Dasman Diabetes Institute, Dasman, Kuwait; 2 Nutrition Unit, Dasman Diabetes Institute, Dasman, Kuwait; 3 Department of Primary Care Health Sciences, University of Oxford, Oxford, United Kingdom; 4 Faculty of Medicine, Kuwait University, Jabriya, Kuwait; Georgia Southern University, UNITED STATES

## Abstract

Although many dietary and lifestyle interventions have been proposed, the concept of total dietary replacement (TDR) to achieve remission of type 2 diabetes in the Gulf region is new. With the high levels of obesity and type 2 diabetes in the region, offering TDR to patients for weight loss and remission of type 2 diabetes would assist in achieving health outcomes. The aim of the current study was to explore and understand remission of type 2 diabetes and TDR from the perspectives of dietitians to identify challenges and recommend solutions for implementation in Kuwait. A qualitative approach utilizing focus groups was chosen to explore the topic. Purposive sampling was used to gain experiences from a diverse sample across primary, secondary, and tertiary specialized diabetes centers. Discussions were audio-recorded and transcribed verbatim. Grounded theory using an iterative approach was applied to analyze the data. Three focus groups with a total of 17 participants achieved data saturation. The sample was varied in terms of workplace and years of experience. The three emerging themes were motivation to use the TDR approach, perceived challenges of TDR, and suggestions to improve and adapt approaches for Kuwait. Dietitians reported that remission of type 2 diabetes is a great motivator for patients to undergo TDR, although various factors were identified that may affect uptake including age, level of education, and social and cultural environment. By understanding dietitians’ perspectives, it has provided insight on views regarding the implementation of TDR to achieve remission in Kuwait and how best to tailor approaches by focusing on patient support needs and adopting a flexible approach.

## Introduction

Obesity is a major world-wide epidemic that is contributing to a variety of diseases, including type 2 diabetes. Diabesity (diabetes associated with obesity) has reached epidemic proportions, both worldwide and in the Middle East [1, 2]. In Kuwait, the prevalence of obesity was reported to be 40.3%, and approximately 8 in 10 adults are living with overweight or obesity [3]. Kuwait has the second highest rates of obesity in the Eastern Mediterranean Region, following Qatar [4]. In terms of diabetes, the prevalence in Kuwait has been found to be 18.8%; this has doubled over the past 20 years [5]. This increasing trend requires immediate attention to reduce the prevalence and address the impact of diabesity on morbidity, mortality and healthcare costs in Kuwait.

Interventions focusing on weight loss are available and wide-ranging, from dietary and lifestyle interventions to pharmacotherapy and surgeries [6–8]. A variety of factors exist that may hinder the uptake and continuation of weight loss interventions; these include personal barriers (such as patients accepting and normalizing their obesity), healthcare system barriers (such as workforce reduction), and community barriers (such as weather conditions and cultural and social norms) [9].

The role of behaviour and psycho-social support is also integral to weight loss services. A systematic review found that patients living with obesity regarded healthcare providers (HCPs) as ambivalent towards them, which affected their willingness to access services [10]. Patients living with obesity also faced stigma and discrimination in daily life, which fed-back into negative psycho-behavioural responses [10]. In the United Arab Emirates, focus groups with 75 women living with obesity and a risk of developing type 2 diabetes found a variety of personal, social, and environmental challenges to managing their weight [11]. Some of these barriers include low motivation, lack of social support, and socio-cultural factors. Another study in Iran with 204 women who were living with overweight or obesity also found stress and depression, as well as education as barriers to the application of weight loss interventions [12].

The implications of weight loss on type 2 diabetes have been recently illustrated, with patients achieving remission of their diabetes after losing weight, using total dietary replacement (TDR), and maintaining their weight loss for a year, as well as stopping their anti-diabetic and hypertension medications [13]. Remission of type 2 diabetes is defined as *“HbA1c of less than 6*.*5% (48 mmol/mol) measured at least 3 months after cessation of glucose-lowering pharmacotherapy”* [8]. This dietary intervention was also recently studied in Qatar in a randomized controlled trial, with 61% of participants achieving remission and 33% achieving normoglycaemia, indicating the feasibility of this intervention in the region [14]. The TDR intervention utilizes formula low-energy diet meal replacement products that are consumed by patients 3–4 times per day for a period of 12 weeks, followed by a 12 weeks structured food reintroduction phase and long-term weight-loss maintenance with behavioural support [15].

Many countries are gradually introducing remission programmes, such as the United Kingdom [16], and with the high prevalence of type 2 diabetes and obesity in Kuwait and the region, it is timely to consider how healthcare professionals perceive the TDR approach for weight loss and remission of type 2 diabetes. In Kuwait, patients with obesity and/or diabetes are referred to dietitians in primary and secondary healthcare facilities for nutrition consultations and health promotion. As little is known about how weight loss advice and diet implementation in Kuwait occurs, the current study aimed to explore and understand the topic of remission of type 2 diabetes and the use of TDR from the perspectives of dietitians to identify challenges and recommend solutions to implement in Kuwait and ultimately, the Gulf region.

## Methods

A qualitative approach was undertaken using focus groups to achieve the study aim. Focus groups were chosen for their time-effectiveness and the ability to explore wide-ranging views by eliciting discussions between participants [17]. Ethical approval was sought from Dasman Diabetes Institute (DDI) in Kuwait where the study took place (RA HM-2018-048).

### Study sample and recruitment

Purposive sampling was employed to fulfil the study objectives; dietitians with experience providing weight loss advice and directly involved with patients with type 2 diabetes met the inclusion criteria. A database of dietitians across Kuwait was used; these were dietitians who attended nutrition workshops at a diabetes institute and agreed to be contacted for future workshops or research. Dietitians were sampled from both DDI and the Ministry of Health (MOH). Dietitians with experience in type 2 diabetes and weight loss were contacted by phone and the study explained; those who met the inclusion criteria were invited to participate. Fifty-three dietitians from across primary, secondary, and tertiary care in various Kuwaiti governorates were invited to participate in the focus groups. Dietitians were provided with a participant information leaflet and signed a consent form before participating in the focus group discussion.

### Data collection

A semi-structured topic guide was developed that was informed by previous literature and the study aim; this was refined after each discussion to ensure emerging topics are explored. The discussions took place at DDI; focus groups were arranged to accommodate the dietitians’ availability from April to May 2019. Each focus group had one moderator (DA) who is an experienced qualitative researcher, and a facilitator taking notes on group behaviour and responses. Discussions were audio-recorded and transcribed verbatim by the facilitator; all the focus groups were in Arabic. Recruitment stopped when data saturation was reached.

### Data analysis and rigour

Principles of grounded theory were employed for data analysis [18]. An iterative approach was taken whereby each discussion was initially analysed and coded to inform the next discussion. It also assisted in determining the point of data saturation [19]. Grounded theory relies on theories emerging rather than being predetermined. Coding is formed from the researcher’s interpretation and refined through constant comparison, wherein data are collected and analyzed constantly to update and improve on future data collection [20, 21]. The qualitative data management software MAXQDA 2018 was used to efficiently manage, code, and retrieve the data [22, 23]. Once coded, these were then organized into themes and sub-themes; a grid was utilized to better compare data across focus groups [24].

To ensure rigour of the methods, various steps were undertaken. These include having a facilitator that took field notes about behavior and interactions to ensure non-verbal communication is not lost [25]. In addition, after each focus group discussion, the moderator and facilitator met and discussed the quality of the discussions and how to improve them. The facilitator transcribed the audio-recording which enhanced the reliability as they are able to understand the discussion and provide notes. Furthermore, the researcher checked all the transcripts against the audio-recordings for accuracy; this also made the researcher familiar with the data and enhanced analysis [26]. The researcher also documented thoughts and personal reflections throughout the data collection and analysis stages for reflexivity purposes. Internal reliability was achieved via the research team discussing the final themes and sub-themes to reach a consensus [27]. To limit translation discrepancies and their impact on the data, the transcripts were kept in the source language [28]. Direct quotes were used to demonstrate the interpretation of the data.

## Results

### Participant demographics

A total of three focus groups took place, with 17 dietitians participating in the discussions. Each focus group had seven, five, and five dietitians respectively. All the dietitians were female, which reflects the state of the workforce in Kuwait where the majority of dietitians are female. The mean age of the dietitians was 30.8 years (SD = 6.7). There was a diversity in terms of workplace, with 3 from primary healthcare centres as well as the Food and Nutrition Association (FNA), 8 from secondary care, and 6 from a diabetes-specific facility. Seventy-six percent are specialised in diabetes, and years of experience was a mean 6.5 years (SD = 6.8).

### Themes

Three major themes emerged from the findings; motivation to use the TDR approach, perceived challenges of TDR, and suggestions to improve and adapt approaches. Table 1 lists the themes, sub-themes, and quotes to illustrate them indicating each participants’ number, age, and speciality for context.

**Table 1:** Themes and sub-themes regarding diabetes remission and using TDR.

*Themes*	*Sub-themes*	*Quotes to illustrate sub-themes*
***Motivation to use TDR***	○ Better glycaemic control ○ Remission and stopping medications ○ Weight loss ○ Easy and fast to prepare products ○ 800 daily calories guaranteed ○ Behaviour and lifestyle change that may be sustained ○ Alternative to bariatric surgery	Remission and stopping medications: *“There’s a goal*, *a higher goal*. *I mean I am ready to do anything just to get rid of my diabetes [talking about what patients may say]” P7 (26 years*, *Diabetes)* 800 daily calories guaranteed: *“Who’s preparing it*, *the salad and chicken*?*” “I know*, *you can’t guarantee your meal will be 800 calories” [discussing eating meals rather than TDR] P13 (30 years*, *Diabetes) and P17 (26 years*, *Diabetes)* Behaviour and lifestyle change that may be sustained: *“But that’s the thing*, *it’s a lifestyle change*. *So*, *when you develop a habit*, *you’re more likely to stick to it later*. *So*, *after the 3 months*, *ok you’re gonna start introducing food but by that stage*, *your stomach is smaller*, *your body has adapted*, *so they won’t go back to their old eating habits” P17 (26 years*, *Diabetes)*
***Perceived challenges of TDR***	○ Dietitian hesitation with patient approach ○ Lack of food ○ Boredom with products ○ Length of intervention (3 months deemed too long) ○ Patient demographics ○ Social and cultural environment ○ Education and awareness level ○ Level of patient motivation and commitment ○ Patient beliefs and preferences	Dietitian hesitation with patient approach: *“I don’t think I’m brave enough to give them [TDR]*, *because it’s 800 calories a day*! *I won’t be able to do it” P16 (28 years*, *Cardiology)* Lack of food: *Because most of them are obese*, *so patients with obesity perceive solid food as essential*. *I mean if it was food*, *it would be better*, *but only liquids*? *That’s difficult” P1 (32 years*, *Diabetes/obesity)* Social and cultural environment: *“There’s another point*, *the diwaniyas [a male social gathering] and gatherings*, *these all play a negative role*. *I mean*, *there are people that want to lose weight*, *they want to change their lifestyle*, *but when I ask them “dinner…” they reply “I can’t change dinner*, *I’m going to diwaniya [social gathering]*. *And if I tell them I can’t eat with them or I have high blood pressure or diabetes*, *they will say that I don’t like their food*. *It’s disrespectful and just isn’t done”” P2 (31 years*, *General nutrition)* Patient demographics/Education and awareness level: *“I think it depends on the age group*, *I mean especially educated people who are well- read and are ready to get rid of their diabetes*, *yes*. *But elderly patients are used to their daily routine and medications and whatever*, *do not go and tell them to change” P17 (26 years*, *Diabetes)* Level of patient motivation and commitment: *“We may have a role to convince people to try it*, *but it all depends on the individual’s motivation*, *is it sustainable*? *He may commit one week and then give up*.*” P2 (31 years*, *General nutrition)* Patient beliefs and preferences: *“Lots of people ask about these things*, *they want Victoza and Saxenda (dietitians laugh) and anything new*, *so we’re lost with them*. *There’s always a new thing and you have to try to convince them…why it isn’t the right way and why what we’re recommending is better*. *So*, *it’s really difficult*.*” P1 (32 years*, *Diabetes/obesity)*
***Suggestions to improve and adapt approaches***	○ Focus on newly diagnosed as more motivated ○ Addition of exercise program ○ Patient engagement ○ Motivation techniques ○ Follow-up frequency ○ Physician involvement ○ Support from HCPs, family, and other patients to meet needs (include psychological support) ○ Dietitian training	Support from HCPs, family, and other patients: *“I think it needs a support group*, *have to have a group*, *so they are always in contact through WhatsApp and they encourage each other and get together” P11 (29 years*, *Diabetes)* Patient engagement/motivation techniques: *“So you*, *when I ask them “tell me what you want*?*” they say “I want this and this” and I reply “ok*, *what’s the easiest to start with*? *And so*, *we start to come to an agreement*, *a shared agreement*. *So*, *what should we start with*? *What’s the priority*? *1 or 2 or 3*? *Maybe their first priority isn’t necessarily my first*, *it may be my tenth…And when we agree on the priorities*, *it’s like a contract between us*, *you know*? *So*, *ok “didn’t we agree on this*? *Didn’t we say this*?*” so they feel better when they trust you*.*” P12 (54 years*, *Diabetes)* Patient engagement/Support *“Let’s say for example you got a group of women*, *all mothers*, *and you put them all together*, *ok*. *When they go home*, *maybe her children*, *maybe her husband they’ll be like “What’s all this [about healthy food]*?*” or “cook us something delicious*. *Don’t cook healthy food”*. *You know this type of talk*, *it can easily break her*. *So*, *you bring in the whole household and educate them together*, *they all learn the same things” P17 (26 years*, *Diabetes)*

#### Motivation to use TDR

Dietitians discussed the topic of remission of type 2 diabetes, which they considered a positive outcome of using TDR. Achieving *better glycaemic control and stopping antidiabetic medications* were stated as powerful motivators for potential patients, alongside the *considerable weight loss*. When discussing products, the majority stated they are e*asy and fast to prepare*, especially for people with a busy lifestyle who do not want to think about what to eat. Furthermore, using the products would make it *easy to verify a total of 800 calories per day*, which would be difficult to ascertain accurately with food. Some dietitians stated that using the TDR approach for 3 months will help patients *change their behaviour and sustain this lifestyle change* for the long-term. It was also mentioned across groups that it would be a great way to market the approach as an *alternative to bariatric surgery*, which many people with obesity are resorting to for weight loss.

#### Perceived challenges of TDR

There were multiple challenges with the TDR approach that emerged from the discussions. A few dietitians felt *hesitant to approach patients* with this intervention, as they feared their reaction. Another reason was that dietitians felt they could not try it themselves, so they were wary of promoting it to their patients. In addition, the *lack of food* was felt as barrier; some TDR approaches depend solely on soups and shakes while others have bars. The dietitians all agreed patients will want some solid food, such as bars, or they will not be able to commit. Furthermore, they felt that the minimal variety of flavours may lead *patients to be bored* easily. The *length of the intervention* (3 months) was also deemed as discouraging to patients as it was felt patients would not be able to tolerate being without solid food for that long.

*Patient demographics*, such as age, gender, and background, were also brought up as playing a major role in accepting TDR. Based on their experiences promoting lifestyle changes to their own patients, the dietitians suggested that younger patients would commit better, with older patients regarded as more difficult to engage with. Regarding gender, male patients were seen to be more committed than female patients. This was due to a variety of factors such as having more family/spousal support and seeing better weight loss results, which increased motivation. In addition, as the majority of dietitians are female, male patients may feel embarrassed if they do not commit and/or show weight loss results. Furthermore, the majority of dietitians stated that female spouses supported their husbands with weight loss, whereas the opposite was not seen frequently in their practice. Background was mentioned in one group, where patients from a Bedouin culture (conservative) were deemed resistant to interventions.

*Social life and the cultural environment* were stated across all groups as a major barrier. Issues like easy access to junk food, lack of family support, social gatherings and obligations where large amounts of food are consumed, and the negative impact of social media were all discussed by dietitians. Following influencers through social media where erroneous weight loss methods and bariatric surgery experiences are shared may negatively influence people. Changes in routine, such as travelling, and fasting in Ramadan, are also setbacks that affect the weight loss journey. Some cultural aspects were seen in a fraction of the population (Bedouin); men do not like their wives eating differently than them, losing weight and changing their appearance, which the dietitians said made the patients’ spouses controlling and unsupportive.

The dietitians all stated that many of their patients with type 2 diabetes have a *lack of education and awareness*. Examples include thinking bariatric surgery will cure their diabetes permanently, eating large portions of healthy food will not make them gain weight, fasting and eating one huge meal will help them lose weight, using fad diets, not counting drinks within their daily calories, and thinking being on anti-diabetic medication is enough. Education is key, and this needs better methods of implementation.

*Patient motivation and commitment* to not only the dietary intervention, but also to attending consultations was deemed a challenge by all the dietitians. Patient behaviour and habits play a role in whether they are motivated and committed to a diet. Dietitians found it difficult changing behaviour, as it was seen to be linked with culture and the patients’ social life. Some are used to eating alongside family members even when they are not hungry, only because it is mealtime, and everyone is eating. The dietitians suggested that support from family and friends also affected behaviour, with a lack in this area enforcing sabotaging behaviour. Changing these behaviours requires a psychological understanding to fix the root of the problem.

*Patient beliefs and preferences* were also discussed as a challenge. Dietitians stated that people with type 2 diabetes are looking for the newest and easiest way to lose weight, whether it is through medications like Saxenda, bariatric surgery, or a fad diet as opposed to a lifestyle intervention that helps them maintain their weight loss. Also, it was stated that patients have to be ready themselves to see a dietitian and make a lifestyle change, where in reality many patients are being pressured to attend by their family or physician referral without any discussion regarding their readiness and motivation. Many patients seen by the dietitians had unrealistic or short-term expectations; examples include losing weight for a wedding or to get pregnant. Patients’ expectations and motivation should be assessed before embarking on a weight loss intervention, such as TDR, to ensure they are committed and to meet their needs. Another finding from the discussions was the majority of patients’ refusal to see a psychiatrist due to stigma associated with mental health issues.

#### Suggestions to improve and adapt approaches

To be able to implement the TDR intervention, the dietitians suggested ways to tailor the intervention to meet the needs of their patients, based on their own clinical experience. It was suggested that focus should be placed on *newly diagnosed patients* who were deemed as more motivated and able to fully commit. Some dietitians recommended the *addition of an exercise programme* to compliment the intervention and to motivate patients who may feel more engaged. Support was regarded as imperative; this was required from HCPs, family members, and other patients on the same journey. A few dietitians suggested having patient support groups, even if through WhatsApp, to encourage each other and share experiences to enhance commitment.

*Patient engagement* needs to be improved. Dietitians discussed how this can be achieved, by having a shared agreement on goals and reaching concordance. It was also stressed that the right relationship was one where there is mutual respect between the dietitian and patient; this fosters trust and therefore the patient is more honest. One solution was using the first consultation for the patient to talk about themselves and their history and what they hope to achieve. Dietitians that do not connect with patients from the first appointment and erect barriers were deemed as unsuccessful by the dietitians. Consultations need to take the individual patient into consideration by assessing their expectations and priorities, their readiness to lose weight, tackling their misconceptions, and possibly involving their family and caregiver in sessions to enhance support.

Dietitians discussed the importance of ensuring patients are motivated and ready to lose weight, and that this needs to be assessed by dietitians before embarking on a dietary intervention. Various methods were brought up to enhance this, such as understanding the patient’s life, history, and their relationship with food to help *tailor motivation techniques*. They should also not be dependent on dietitians as they need to sustain their lifestyle changes on their own. One dietitian lets patients evaluate themselves on the first day, then compares again after a month to see how far they have come. Others give incentives such as how their weight loss is leading to better blood glucose control, lowering medication doses or stopping them, and decreasing hospital admissions. Some relied on fear tactics to motivate patients to commit, while others try and target patient behavior.

*Frequent follow-up* was recommended as it would help facilitate commitment and weight loss, with 2 weeks between appointments suggested as standard. However, it was highlighted that not all patients can attend due to work constraints, and this was one reason they did not commit. Dietitians proposed that patients who frequently missed appointments should be focused on.

*Physicians have an important role* and dietitians discussed how they were ignorant of the importance of the dietitian and lifestyle interventions. Most physicians were seen as focusing on medications and increasing doses to control blood glucose. This is also one reason why referral to dietitian consultations in general is low. As patients place physicians above dietitians, physicians are able to encourage patients to visit a dietitian and try TDR.

The *need for psychological assessment and support* was echoed across all focus groups. Many patients are depressed or are emotionally binge eating. Dietitians felt unable to deal with psychological issues, such as depression, to help people lose weight, and there is no mental support for patients. They felt there is a need for a psychiatrist for obesity and diabetes, which is not available. Support for dietitians to deal with such cases is also non-existent, even though most of their consultations deal heavily on psychology. In addition, not all dietitians felt comfortable referring to a psychiatrist, and those who do say patients refuse to go due to stigma and other reasons such as using this information for divorce proceedings.

All the participating dietitians expressed that *dietitian training needed improvement* regarding implementing TDR. By improving dietitian education with continuing professional development programmes to enhance their knowledge and training, specifically focusing on psychological interventions, this may help in improving their approach and motivating patients. Dietitians were all in agreement that they needed psychological training, such as cognitive behavioral therapy, to help them meet patients’ needs.

## Discussion

Dietitians may encounter a variety of challenges and facilitators when trying to provide education regarding remission of type 2 diabetes and interventions to attain this, like TDR; the current findings have shed light on what these are in the Kuwaiti context. To our knowledge, this is the first study to approach this subject in Kuwait and the Gulf region. Although there were some discrepancies in views, certain aspects were shared by the majority of dietitians in this study.

Dietitians agreed that attaining remission of type 2 diabetes was a positive outcome of using TDR; nevertheless, there is a need to adapt the approach to overcome the challenges identified. Age, flexibility of intervention, and social and cultural environment were discussed as barriers for acceptability of the intervention. Previous literature supports socio-cultural norms as a challenge [9, 11, 29, 30]. In Kuwait, social gatherings and obligations are part of the culture, and for the approach and intervention to be successful, the patients’ social environment needs to be understood and the correct support provided. Social pressure may exacerbate the patient’s adherence to a dietary intervention, and patients with the ability to cope with eating differently from those around them adhere better [29].

With the higher obesity prevalence in the region and the resultant normalization of obesity [31] and greater uptake of surgical interventions for weight loss [32], dietitians may feel dietary interventions would not be acceptable, which is the case with the dietitians in the current study. Furthermore, less frequent follow-up and unavailability of outcome data also play a role. Dietitians also lacked the proper training to deliver interventions achieving remission. In a previous study from the UK, patients highlighted the importance of the dietitians’ approach and behavior on their commitment [33]. This included tailoring the consultation (prescriptive Vs. nonprescriptive), establishing a partnership, supporting behavior change, and listening to their needs. This was similarly reflected in the current findings, although behavior change was difficult for the dietitians as they lacked the tools and knowledge to implement this confidently. Behavior change is deemed essential when developing effective dietary interventions [34]. One study surveying dietitians found that they felt inadequate regarding behavior change techniques and needed training [35].

Previous literature has indicated that healthcare professionals may not always be focusing on the barriers encountered by their patients, or share the same priorities in terms of care [29, 36]. The current findings show that dietitians in our sample are striving to identify their patients’ needs and establishing a partnership with them, with agreement on shared goals and expectations, which is reassuring, as these assist in establishing the correct foundation for interventions to be successful [37]. Nevertheless, this may not be representative of the profession as a whole in Kuwait and in the Gulf region. Dietitians require professional support to achieve optimal patient care, as they are limited by workplace restrictions and processes [38].

Studies using surveys to gain insight into HCP views regarding meal replacement products and their use in very low energy diets within their practice showed there was some concern regarding the safety of these products and the long-term effectiveness of this intervention [39, 40]. In addition, adherence by patients was identified as a barrier, as was the need for comprehensive training. Another study using interviews with HCPs following the TDR intervention in the UK highlighted that some HCPs felt apprehensive towards stopping their patients’ anti-diabetic and anti-hypertensive medications [41]. These concerns and barriers were also discussed by the dietitians in the current study; these points need to be addressed in any future education and training of HCPs delivering this intervention, as their personal beliefs, perceptions, and confidence in the intervention are integral towards adopting it.

Dietitians highlighted that psychological support is lacking and this issue needs addressing, as they felt inadequate in that regard. Stigma towards psychological support and the lack of accessible support in primary healthcare is known in Kuwait [42]. Stigma was attributed to cultural factors, such as familism and the importance of reputation [43]. This was similarly professed by the dietitians in this study, and it was suggested that dietitians be trained to perform evaluations to overcome this challenge and ensure patient outcomes are achieved.

The DIADEM study demonstrated that the TDR intervention is feasible in Qatar, which may translate to the Kuwaiti population, although North African participants made up a higher proportion than Middle Eastern participants which may indicate the reduced uptake of the intervention in patient population from the Gulf region [14, 44]. This study also included an exercise component, which was advocated by the dietitians in the current study as a motivator.

Frequency of follow-up and support are deemed important for the success of the intervention, with a biweekly follow-up echoed across studies [13, 14], although one trial took a more patient-centered approach with weekly support with a counselor either by phone or face-to-face [45].

Results highlight the enthusiasm to use lifestyle interventions to achieve remission, but that the intervention should be adapted by focusing on patient support needs, with a flexible approach that takes culture and social life into consideration. By understanding dietitians’ thoughts, an adapted approach can be implemented to meet the needs of the Kuwaiti population, which can also be translated to the Gulf region. Furthermore, comprehensive dietitian training should be implemented before piloting the TDR intervention.

### Strengths and limitations

Although the sample size was small, the dietitians were sampled from across healthcare settings in Kuwait, and therefore the findings may be generalizable to primary and secondary healthcare in the public sector. Unfortunately, private sector services were not included, as they were not within the scope of the study. Nevertheless, some of the dietitians were recruited from a diabetes-specific facility, which reflects similar care as that seen in the private sector. Another strength is that the researcher is of the same gender and age group as the dietitians, and is a healthcare professional which aided the data collection and analysis stages [27]. Countries within the Gulf region share similar traditions and culture, but it would be beneficial to gain perspectives across countries in the region for comparison and generalizability of findings.

## Conclusions

Findings have identified dietitians’ perspectives on the acceptability of using TDR to achieve diabetes remission in Kuwait. Various challenges were identified and suggestions to tailor approaches to meet patient needs were provided. This work has assisted in setting a framework to pilot a TDR intervention in Kuwait to assess the acceptability from the patients’ views. Understanding patient and HCPs’ perspectives and preferences is imperative to develop and deliver an acceptable intervention to achieve remission of type 2 diabetes in Kuwait and the region. By meeting patient and dietitians’ needs, health outcomes can be achieved and satisfaction with care can be improved.

## Supporting information

S1 File Topic guide for healthcare professionals focus groups. (PDF)
