# Supplementary material for: Remission of type 2 diabetes: Perspectives of dietitians in Kuwait
Source: PLoS One. 2022 Oct 27;17(10):e0276679. doi: 10.1371/journal.pone.0276679 (PMC9612548; doi:10.1371/journal.pone.0276679)
Supplement: S1 File — (PDF) [file pone.0276679.s001.pdf]

<u>Topic guide for health care professionals focus group</u>

<u>Ice breaker:</u> what type of patients do you usually see in your practice?

1. What are your past experiences providing weight management education/advice to patients with type 2 diabetes?

2. What were the most successful weight-loss method approaches you tried? And why?

3. What were the most challenging weight-loss method approaches you tried? And why?

4. What are your thoughts on intermittent fasting?

5. What support do you think patients need to help them maintain weight-loss?

6. What do you think about the feasibility of implementation of a low-calorie total diet replacement that can cause remission of diabetes in practice? (After explaining to them what the intervention entails)

7. What are your suggestions towards encouraging patients to use the TDR intervention to achieve remission?
